# Mapping the literature of health care management: an update

**DOI:** 10.5195/jmla.2021.1121

**Published:** 2021-07-01

**Authors:** Amber T. Burtis, Susan M. Howell, Mary K. Taylor

**Affiliations:** 1 amber.burtis@siu.edu, Southern Illinois University Carbondale, Carbondale, IL; 2 showell@lib.siu.edu, Southern Illinois University Carbondale, Carbondale, IL; 3 mtaylor@lib.siu.edu, Southern Illinois University Carbondale, Carbondale, IL

## Abstract

**Objective:**

This study aims to identify the core journals cited in the health care management literature and to determine their coverage in the foremost bibliographic databases used by the discipline.

**Methods:**

Using the methodology outlined by the Medical Library Association's Nursing and Allied Health Resource Section (NAHRS) protocol for “Mapping the Literature of Nursing and Allied Health Professions,” this study updates an earlier study published in 2007. Cited references from articles published in a three-year range (2016–2018) were collected from five health care management journals. Using Bradford's Law of Scattering, cited journal titles were tabulated and ranked according to the number of times cited. Eleven databases were used to determine coverage of the most highly cited journal titles for all source journals, as well as for a subset of practitioner-oriented journals.

**Results:**

The most highly cited sources were journals, followed by government documents, Internet resources, books, and miscellaneous resources. The databases with the most complete coverage of Zone 1 and 2 were Scopus, Web of Science Core Collection, and PubMed, while the worst performing databases were Health Business Elite, ABI/Inform, and Business Source Complete.

**Conclusions:**

The literature of health care management has expanded rapidly in the last decade, with cumulative citations increasing by 76.6% and the number of cited journal titles increasing by nearly 70% since the original study. Coverage of the core journals in popular databases remains high, although specialized health care management and business databases did not perform as well as general or biomedical databases.

## INTRODUCTION

Health care management (HCM) involves the administration, planning, directing, and coordination of medical and health services. Medical and health services managers work in a variety of different settings, such as health care facilities, including hospitals and nursing homes, and group medical practices. A bachelor's degree is the minimum requirement for entering the field, but master's degrees also are common. Medical and health services managers typically have some work experience in an administrative or a clinical role in a hospital or other health care facility and must be knowledgeable about health care laws, regulations, and technology. Common degrees for medical and health services managers are typically health administration, health management, nursing, public health administration, or business administration [1]. As of 2018, there were approximately 406,100 medical and health services managers in the United States. The Bureau of Labor Statistics 2018-28 Job Outlook predicts better-than-average growth of 18% (or 71,600 new jobs by 2028) [2].

There has been drastic change in the HCM field in the last decade. The American Recovery and Reinvestment Act of 2009 injected billions of dollars into health services research–related spending, including the study of the comparative effectiveness of health care treatments and health information technology infrastructure. One year later, the Affordable Care Act of 2010 put significant investments into health services research with the creation of the Patient-Centered Outcomes Research Trust Fund and Institute. This investment in the nation's health care spending, which catalyzed the advancement of technology such as electronic health records, along with developments in big data analytics, have moved the HCM field toward a more data-rich research environment [3].

This study updates a study published in 2007, in which the authors used bibliometric methods to map the literature of health care management; in the 2007 study, the authors found that HCM is a multidisciplinary field, drawing from research in health services or health policy, biomedicine, health care administration, and business [4]. Williams et al. argue that bibliometrics (e.g., journal impact factor and journal-rating and ranking lists) play an important role in identifying the core journals of HCM since it is a “multidisciplinary field which is housed in a broad variety of schools and colleges … [that] differs from other disciplines in that it brings a diverse, multidisciplinary faculty together to investigate and teach about the health care industry” [5]. For this reason, mapping patterns of citation to a variety of disciplinary scholarly journals is an especially apt approach for examining a multidisciplinary field like health care management. Previous studies evaluating publication outlets in HCM have identified core journals based on faculty ratings of perceived quality, relevance, or both [6–11] or bibliometric studies [12, 13]. In addition to analyzing the difference in the literature, the researchers aim to offer collection librarians useful information upon which to base decisions regarding which publications and indexes are essential. As in the previous study, the focus is on general HCM in US journals.

## METHODS

For purposes of comparison, the same five source journals from the 2007 study were used: *Health Affairs* (HA), *Health Care Management Review* (HCMR), *Health Services Research* (HSR), *Journal of Healthcare Management* (JHM), and *Medical Care Research and Review* (MCRR) [4]. They were analyzed using the methods set forth in ‘‘Mapping the Literature of Nursing and Allied Health Professions Project Protocol'' from the Medical Library Association's Nursing and Allied Health Resource Section [14]. These journals still met the source selection criteria of the protocol, which includes recommendations to ameliorate the problem of implicit bias. The protocol states that “the source journals selected should cover the field comprehensively.” Three journals (HA, HSR, and MCRR) are health services policy research oriented, whereas the other two (HCMR and JHM) are more management and practitioner oriented. It also suggests that journals from professional associations in the field be selected because, as benefits of membership, they “may be the [journals] that [are] most accessible and most highly read by practitioners in that specialty/discipline.” Professional association journals included as sources in this study included HA (AcademyHealth), HSR (AcademyHealth), and JHM (American College of Healthcare Executives). The protocol also recommends utilizing the opinions of faculty and librarians in the field. Recent surveys of health care research and management faculty members and researchers have affirmed the importance of these five journals [9–11]. The participants in Borkowski et al.'s study ranked these journals as the top five most relevant health-related titles as potential publishing sources for HCM research [11]. Meese et al. found that all five titles were among the top twenty most influential journals in HCM according to the opinion of international experts, with HCMR (rank=1), HA (rank=2), HSR (rank=4), and JHM (rank=6) in the top ten [10]. Menachemi and DelliFraine surveyed health administration faculty members and found that all five of this study's source journals were ranked in the top ten health administration journals, with HA, HSR, HCMR, and JHM in the top five [9]. In addition, all five journals are also listed as core journals on the authoritative Medical Library Association Public Health/Health Administration Core Public Health Journal Project list [15].

References from research articles in the five source journals published from 2016 to 2018 were entered into a Microsoft Access database. Letters to the editor, editorials, historical reprints, and brief items were excluded. The data collected for each cited reference included the year of publication, name of the source journal, and format. Each cited reference was assigned a unique identifier and coded as one of the following formats: journal article, book, government document (print or online, published at the local, regional, national, and international level), Internet resource (not government sponsored), or miscellaneous (e.g., dissertations, software, etc.). Previous “Mapping the Literature of Nursing and Allied Health Professions” project protocols only counted print and PDF versions of government resources as government documents, but this study counts any print and any Internet-based government resource as a government document. Journal titles were collected and journals with title changes were combined under the most recent title. The National Library of Medicine (NLM) catalog was used to trace journal title changes and normalize journal titles.

References were sorted by format and year of publication, and the cited journal titles were isolated. Bradford's Law of Scattering [16] was then applied, which predicts that “there are a few very productive periodicals, a larger number of more moderate producers, and a still larger number of constantly diminishing productivity.” The cited journal titles were separated into three zones, each containing approximately a third of the total cited journal references. Zone 1 contained the highly productive titles, Zone 2 contained moderately productive journals, while Zone 3 contained the least productive sources.

Coverage of cited journal titles from Zones 1 and 2 was determined from online title lists for the following databases: ABI/Inform, Business Source Complete, Cumulative Index to Nursing and Allied Health Literature (CINAHL) Complete, EMBASE, Health Business Elite, ProQuest Health Administration, PubMed, Science Citation Index, Scopus, Social Sciences Citation Index, and Web of Science Core Collection (which includes Science Citation Index and Social Sciences Citation Index in addition to Humanities Citation Index). Coverage in the database was noted as “Yes,” “No,” or “Ceased.” To determine the exclusive coverage of these databases, MEDLINE citations were excluded from the results for EMBASE and Scopus. Scopus was the only additional database evaluated in this study as compared to the 2007 study.

Statistical analysis using Welch's *t* test was performed to determine whether there was a statistically significant change in the number of articles per journal issue since the 2007 study. The average and standard deviation for the number of articles per issue was calculated in Excel, and an online *t* test calculator (graphpad.com) was used to calculate the *t* value, *df*, and *p* values reported in Table 2.

## RESULTS

In total, 51,758 cited references were collected: 70.4% were journal articles, 12.5% were government documents, 9.6% were Internet sources, 4.1% were books, and 3.5% were miscellaneous (Table 1). *Health Affairs* citations (n=22,141) accounted for the largest proportion of the total citations (42.8%).

**Table 1:** Cited resource types by source journal and frequency of citations for all source journals

Cited resource type	No. citations in source journals						
	HA	HCMR	HSR	JHM	MCRR	Total	Frequency
Books	564	427	686	216	223	2,116	4.1%
Journal articles	14,051	3,177	13,610	2,130	3,470	36,438	70.4%
Government documents	3,681	104	2,069	236	357	6,447	12.5%
Internet resources	3,154	96	1,003	368	331	4,952	9.6%
Miscellaneous	691	57	848	67	142	1,805	3.5%
Total	22,141	3,861	18,216	3,017	4,523	51,758	100.0%

HA = *Health Affairs*; HCMR = *Health Care Management Review*; HSR = *Health Services Research*; JHM = *Journal of Healthcare Management*; MCRR = *Medical Care Research & Review*

The increase in the number of articles per issue since the 2007 study was statistically significant (*p*≤0.05) for *Health Care Management Review, Health Services Research*, and *Journal of Healthcare Management* (Table 2). Although the number of issues of *Health Affairs* increased from 22 to 36 because the journal publication schedule changed from bimonthly with additional online articles to monthly, the number of articles per issue did not significantly change as a result.

**Table 2:** Source articles published: 2002–2004 and 2016–2018

	Source Articles Published									
	HA		HCMR		HSR		JHM		MCRR	
Source year	2002–2004	2016–2018	2002–2004	2016–2018	2002–2004	2016–2018	2002–2004	2016–2018	2002–2004	2016–2018
Number of articles	471	758	84	100	269	507	98	114	78	103
Number of issues	22	36	12	12	21	18	18	18	16	18
Average articles per issue	21.41	21.06	7.00	8.33	12.81	28.17	5.44	6.33	4.88	5.72
Standard deviation*	9.50	2.89	1.48	0.89	2.75	15.93	0.86	1.19	1.67	1.23
*t* value†		0.168		2.668		−4.04		−2.572		1.653
*df*		23		18		17		30		27
*p* value		0.868		0.016		0.001		0.015		0.110

* STD of number of articles

† Using two-tailed test, equal variances not assumed

HA = *Health Affairs*; HCMR = *Health Care Management Review*; HSR = *Health Services Research*; JHM = *Journal of Healthcare Management*; MCRR = *Medical Care Research & Review*

The majority of cited references (62.7%; n=32,433) were from the eight-year time period from 2010 to 2018 (Table 3). Government documents and Internet resources accounted for 60.0% of the cited references from the 2016–2018 time period, and only 10.8% were from journals. The currency of cited references increased since the original study. Excluding items for which dates were not available, this change was statistically significant (χ^2^ = 497.134, *df* =6, *p* = 0.00).

**Table 3:** Cited resource types by publication year periods

Publication year	Book No.	%	Gov No.	%	Int No.	%	Jour No.	%	Misc No.	%	All No.	%
2016–2018*	80	3.8%	1,838	28.5%	1,559	31.5%	3,948	10.8%	250	13.9%	7,675	14.8%
2010–2015	619	29.3%	3,243	50.3%	2,689	54.3%	17,229	47.3%	978	54.2%	24,758	47.8%
2004–2009	463	21.9%	618	9.6%	395	8.0%	8,538	23.4%	322	17.8%	10,336	20.0%
1994–2003	540	25.5%	344	5.3%	97	2.0%	4,987	13.7%	133	7.4%	6,101	11.8%
1984–1993	213	10.1%	45	0.7%	7	0.1%	1,146	3.1%	29	1.6%	1,440	2.8%
1974–1983	117	5.5%	11	0.2%	0	0.0%	385	1.1%	17	0.9%	530	1.0%
Pre-1974	81	3.8%	11	0.2%	0	0.0%	200	0.5%	40	2.2%	332	0.6%
No date	3	0.1%	337	5.2%	205	4.1%	5	0.0%	36	2.0%	586	1.1%
Total	2,116	100.0%	6,447	100.0%	4,952	100.0%	36,438	100.0%	1,805	100.0%	51,758	100.0%

* Includes materials in press

Book = Books; Gov = Government documents; Int = Internet; Jour = Journals; Misc = Miscellaneous; All = All resource types

The 36,438 journal article citations from all five source journals represent 2,995 different journal titles (Table 4). There was an almost doubling of cumulative journal citations since the 2007 study, from 18,393 to 36,438, an increase of 98.1%. The total number of journal titles also increased drastically in that time period, from 1,766 to 2,995 (an increase of more than 69.6%).

**Table 4:** Distribution by zone of cited journals and references

	All journals					HCMR and JHM only				
	Cited journals		Cited journal references			Cited journals		Cited journal references		
Zone	No.	%	No.	%	Cumulative total	No.	%	No.	%	Cumulative total
1	10	0.3%	12,181	33.4%	12,181	16	1.5%	1,785	33.6%	1,785
2	105	3.5%	12,128	33.3%	24,309	99	9.1%	1,807	34.0%	3,592
3	2,880	96.2%	12,129	33.3%	36,438	972	89.4%	1,715	32.3%	5,307
Total	2,995	100.0%	36,438	100.0%		1,087	100.0%	5,307	100.0%	

Zone 1 contained 10 journals that accounted for 33.4% of the cited journal references (Table 4). These Zone 1 journals included a mix of health services research and policy journals (e.g., *Health Affairs, Health Services Research, Medical Care, Journal of Health Economics*), biomedical journals (e.g., *JAMA, New England Journal of Medicine, JAMA Internal Medicine, Annals of Internal Medicine, Journal of General Internal Medicine*), and a public health journal (e.g., *American Journal of Public Health*).

Zone 2 contained 105 journals and accounted for 33.3% of the cited journal references (Table 4). Using Bradford's Law of Scattering, n for this study was calculated as 10.5 (105 journals in Zone 2/10 journals in Zone 1). Bradford's Law predicts that the number of Zone 3 journals in this study should be 1,050 (10.5 × 10). However, there were 2,880 journals in Zone 3, an excess of more than 1,800 journals [16].

More than half of the databases covered 100% of titles in Zone 1 (Supplemental Table 5). Scopus indexed all but one of the journals in Zone 1 and 2 (99.1%), but no single database indexed all of the journals. Web of Science Core Collection had better coverage of Zone 1 and Zone 2 journals (97.4%) than PubMed (82.5%), CINAHL (77.2%), and Science Citation Index (77.2%). The worst-performing databases were Health Business Elite (13.2%), ABI/Inform (21.9%), and Business Source Complete (25.4%).

Supplemental Table 6 highlights the distribution and database coverage scores for the practitioner-oriented journals in this study (*HCMR and JHM*). Scopus and Web of Science Core Collection indexed 100% of the journals in Zone 1. Scopus had the best coverage of Zones 1 and 2 (93.9%) and Web of Science Core Collection had better coverage of Zone 1 and Zone 2 journals (90.4%) than PubMed (69.6%), CINAHL (66.1%), and Social Sciences Citation Index (64.3%). The worst performing databases for Zone 1 and Zone 2 combined were Health Business Elite (20.9%), ABI/Inform (38.3%), and Business Source Complete (41.7%).

## DISCUSSION

The literature of HCM has expanded rapidly in the period between the two studies. Cumulative citations of all resource types increased 76.6% (from 29,305 to 51,578). The fact that there were also more source articles overall, especially for *Health Services Research* and *Health Affairs*, might offer one explanation for the increased number of citations. Another reason for the increased number of citations might be the increase in review articles, which tend to have more citations, from a total of 20 in the first study to 41 in the second.

Cumulative journal citations almost doubled between the two studies, from 18,393 to 36,438, an increase of 98.1%. The trend toward an increased number of cumulative citations of any resource type and to journal articles, specifically, can be seen in other updates of mapping studies using the “Mapping the Literature of Nursing and Allied Health Professions” project protocols [17–22]. The 2019 update [17] of a 1999 dental hygiene mapping study [18] reported an increase of 134.3% in the number of total citations for the two source journals in common and a 157.7% increase in citations to journals. The 2011 update [19] of a 1997 physical therapy mapping study [20] found an increase of 47.7% in the number of total citations for the one source journal in common and an increase of 74.4 % in citations to journal articles. The 2010 update [21] of a 1997 health education mapping study [22] found an 80.1% increase in the total number of citations for the four source journals in common and an increase of 103.5% in the number of citations to journals.

Journal citations grew in their share of the total citations in the HCM literature, from 62.8% (n=18,393) in 2007 to 70.4% (n=36,438) in this study. This increase could be explained by a reordering of the frequency of citations to other resource types. For instance, in their update of the dental hygiene literature, Watwood and Dean found that book citations decreased from 18.1% of total citations in 1999 to 5.1% in 2019 and miscellaneous citations decreased from 7.4% in 1999 to 1.6% in 2019 [17]. The authors suggest that this change was because researchers now browse the web for information they used to find in books and because miscellaneous resources may have migrated to the web. We found a similar trend, with book citations decreasing from 16.5% (n=4,825) of total citations in 2007 to 4.1% (n=2,116) in the current study and miscellaneous citations decreasing from 8.9% (n=2,609) to 3.5% (n= 1,805). We concur with that explanation, as we also saw an increase in Internet resources from 3.4% (n=1,004) in 2007 to 9.6% (n=4,952) in the current study, as well as an increase in government documents (which are largely online) from 8.4% (n=2,474) to 12.5% (n=6,447).

In the past mapping protocols, only government web-based citations that were PDFs counted as government documents. The current study included 2,284 government documents with PDF anywhere in the URI out of a total of 6,447 government documents. This could explain part of the increase in government documents in the study, although there was also still an increase in print (n=2,474) and non-PDF web-based government documents (n=3,690). The increase in the number of government documents could be due to the increased availability of online resources.

Just as in the 2007 study, where 84.1% (n=24,633) of the cited references were from the health services or health policy research source journals (*Health Affairs, Health Services Research*, and *Medical Care Research and Review*) over the practitioner-oriented source journals (*Journal of Healthcare Management* and *Health Care Management Review*), 86.7% (n=44,880) of the cited references in this study were from those same health services or health policy research source journals.

In the 2007 study, four health services research and policy titles and two biomedical titles made up Zone 1. Citations to health services research and policy titles (n=4,076, 67.3%) outweighed the citations to biomedical titles (n=1,978, 32.7%). In this study, the titles in Zone 1 were evenly split with five health services research and policy titles and five biomedical titles. While citations to health services research and policy titles (n=7,199, 59.1%) still outweighed the citations to biomedical titles (n= 4,982, 40.9%), the gap had narrowed from 34.6% to 18.2%. This may be due to changes in government funding and focus mentioned in the introduction to this article. The Recovery and Reinvestment Act of 2009 and the Affordable Care Act of 2010 emphasized research that focused on the analysis of the comparative effectiveness of health care therapies and patient outcomes [3]. This type of research requires data from biomedical fields and thus increased use of biomedical journals. It may also be conjectured that there has been an increase in the number of policy-focused articles in biomedical journals as a result of the emphasis on outcomes research and comparative effectiveness, such as those that have long been included in *New England Journal of Medicine, JAMA*, and *BMJ* [23]. Additionally, this may be because health care management, like other health-related fields, has increased its focus on evidence-based research and requires data from biomedical fields to back up policy and management research recommendations (e.g., recommendations or comments on the effectiveness for one type of surgery or public health intervention over another).

In the original study, six of the eleven titles in Zone 1 of the practitioner-oriented source journals were health services research and policy journals, four were general management titles, and only one was a medical journal. Citations to health services research and policy titles (n=636) outweighed the citations to biomedical titles (n=90) and the general management titles (n=293). In this study, there were nine health policy or health services journals and three management journals. Note that *New England Journal of Medicine* is a clinical medical journal known also for articles about health policy [23]. Citations to health services research and policy titles (n=920) outweighed the citations to biomedical titles (n=349) and the general management titles (n=253).

The journal citations in this study sample represent 2,995 different journal titles (Table 4). The total number of journal titles increased drastically in the time period between the two studies, from 1,766 to 2,995 (an increase of greater than 69.6%). This was perhaps due to an increased emphasis on evidence-based practice, although it may also be due to an overall increase in the number of academic journals being published. Zones 1 and 2 were more widely dispersed in the current study, with Zone 2 doubling in size since the original study and Zone 1 increasing from six to ten titles. Zone 3 had 1,800 more journals than Bradford's Law predicts, pointing to an extremely diverse and wide-ranging set of journals that make up the literature of health care management.

Updates of other mapping studies have also found the increase in the dispersion of titles in the zones to be of interest. Watwood and Dean found an increase in the total number of journal titles in their update of the dental hygiene literature [17] and suggest it “might be attributed to the growing utilization of online-only journals.” Although electronic journals were available before the advent of the World Wide Web, it is true the source journals in the earliest NAHRS-allied health mapping studies pre-date the growth of online journals made increasingly possible by the protocols of the Web and the development of browser client programs such as NCSA Mosaic in the early 1990s [24, 25].

Another source of dispersion may come from the rise of freely available bibliographic databases that cover the health care mapping literature. Watwood suggests that “the increasingly ‘long tail' of journal titles [in Zone 3] may come partly from the use of Google Scholar” [26]. This might explain increases after 2004, the year that Google Scholar was introduced. However, the move to direct researcher access to the medical literature was already being facilitated by debuts of PubMed and Grateful Med in the late 1990s, which provided free access to MEDLINE and other NLM/NCBI database citations via a web format that had previously resided behind a paywall and could only be accessed by commercial bibliographic utilities [27, 28]. The increase in dispersion may also be due to the accelerated rise of open access journals, funder mandates, and institutional repositories.

The databases with the best coverage of Zone 1 titles and overall were the Web of Science Core collection and Scopus, both of which had 100% coverage. The worst-performing databases in Zones 1 and 2 were, much like the 2007 study, the specialized business and HCM databases Business Source Complete, Health Business Elite, and ABI/Inform. In sum, librarians serving health care administration researchers might do better subscribing to general databases such as Scopus or Web of Science and consider adding CINAHL Complete, in addition to providing access to and linking out to their journal collections via PubMed.

There were some differences between the two studies. First, Scopus was not evaluated in the original study. In addition, database coverage was determined in the first study by searching databases for the number of articles indexed and in this study by searching journal title lists provided by publishers to determine coverage, as the protocol had changed in the intervening time period. This study also has some limitations. The validity of citation analysis depends on the accuracy of source citation data. Data were reviewed for outliers, but not citation by citation.

## CONCLUSIONS

The literature of HCM has expanded in the decade between this study and the original, with cumulative citations increasing by 76.6% and the number of cited journal titles increasing by 69.6%. The trend toward an increased number of cumulative citations of any resource type and to journal articles, specifically, can be seen in other updates of mapping studies. Updates of other mapping studies have also found an increase in the dispersion of titles in the zones. This study proposes several possible reasons for this phenomenon, including the growing utilization of online-only journals, the rise of freely available bibliographic databases (e.g., Google Scholar), and the accelerated rise of open access journals, funder mandates, and institutional repositories.

Coverage of the core journals in popular databases remains high, although HCM and business databases do not perform as well as general or biomedical databases. Libraries serving health care administration researchers should prioritize subscribing to general databases such as Scopus or Web of Science and consider adding CINAHL Complete in addition to providing access to and linking out to their journal collections via PubMed.

## Data Availability

Data associated with this article are available in the authors' institutional repository at <https://opensiuc.lib.siu.edu/morris_data/1/>.

## SUPPLEMENTAL FILES

**Supplemental Table 5** Distribution and indexing coverage of cited journals in Zone 1 and Zone 2

**Supplemental Table 6** Distribution and indexing coverage of cited journals in Zone 1 and Zone 2 from practitioner-oriented journals: *Health Care Management Review* and *Journal of Healthcare Management*
